# Prevalence and Predictors of Malnutrition Risk among Post-Stroke Patients in Outpatient Setting: A Cross-Sectional Study

**DOI:** 10.21315/mjms2020.27.4.7

**Published:** 2020-08-19

**Authors:** Hui Jie Wong, Sakinah Harith, Pei Lin Lua, Khairul Azmi Ibrahim

**Affiliations:** 1 School of Nutrition and Dietetics, Faculty of Health Sciences, Universiti Sultan Zainal Abidin, Gong Badak Campus, Terengganu, Malaysia; 2 Faculty of Pharmacy, Universiti Sultan Zainal Abidin, Besut Campus, Terengganu, Malaysia; 3 Neurology Unit, Department of Medicine, Hospital Sultanah Nur Zahirah, Jalan Sultan Mahmud, Terengganu, Malaysia

**Keywords:** stroke, malnutrition risk, prevalence, cross-sectional study, Malaysia

## Abstract

**Background:**

The present study examined the prevalence and predictors of malnutrition risk among post-stroke patients.

**Methods:**

Post-stroke patients who attended the outpatient clinics in three hospitals of Peninsular Malaysia were enrolled in the study. The risk of malnutrition was assessed using the Malnutrition Risk Screening Tool-Hospital. Data including demographic characteristics, clinical profiles, dietary nutrients intake, body mass index (BMI) and hand grip strength were collected during the survey. The crude odds ratio (OR) and adjusted odds ratio (AOR) were reported for univariate and multivariate logistic regression analyses, respectively.

**Results:**

Among 398 patients included in the study, 40% were classified as high-risk for malnutrition. In the multivariable logistic regression, tube feeding (AOR: 13.16, 95% confidence interval [CI]: 3.22–53.77), loss of appetite (AOR: 8.15, 95% CI: 4.71–14.12), unemployment (AOR: 4.26, 95% CI: 1.64–11.12), wheelchair-bound (AOR: 2.23, 95% CI: 1.22–4.09) and BMI (AOR: 0.87, 95% CI: 0.82–0.93) were found to be significant predictors of malnutrition risk among stroke patients.

**Conclusion:**

The risk of malnutrition is highly prevalent among post-stroke patients. Routine nutritional screening, identification of risk factors, and continuous monitoring of dietary intake and nutritional status are highly recommended even after the stroke patient is discharged.

## Introduction

Malnutrition is common among stroke survivors, with its prevalence ranging from 6.1%–62% (1). It is important to identify and treat malnutrition because it is often linked to poor functional outcomes, increased complications, long hospital stay, increased mortality, and higher hospitalisation costs (2–4). However, examining the nutritional status of a stroke patient is not always a straightforward task due to the absence of a gold standard for nutritional assessment (1). A previous review study has defined malnutrition based on a single or combination of anthropometric data (i.e. body mass index (BMI), calf circumference, mid-upper arm circumference, triceps skinfold, or changes in body weight) and clinical laboratory or nutritional risk screening tools (1). Although BMI is often recommended in the diagnosis of malnutrition (5), accurate anthropometric measurements among stroke patients with disability remain a great challenge. Previous studies have reported that almost half of stroke patients had not undergone routine BMI assessment (6, 7). In addition, recent studies have suggested the use of a validated nutritional screening tool as a more rapid and simpler way to diagnose malnutrition (8) or predict poor functional recovery (9, 10) and clinical outcomes (11–13) among stroke patients.

While there are accumulative pieces of evidence regarding the prevalence of malnutrition risk using nutritional screening tools among stroke patients during acute or subacute phase (9, 10, 14–16), little is known about the condition during rehabilitation or in an outpatient setting (17–19). Moreover, most of the literature has specifically focused on elderly stroke population (9, 10, 14, 18). Similarly, publications on dietary intake (17, 20, 21) and predictors of malnutrition risk (19, 22, 23) among post-stroke patients after discharge are scarce. Therefore, the present study examined the prevalence and predictors of malnutrition risk among post-stroke patients attending outpatient clinics.

## Methods

### Study Design and Setting

This cross-sectional observational study was conducted in the outpatient clinics of three hospitals located in the East Coast region of Peninsular Malaysia between May and August 2019. All stroke patients who visited the outpatient clinics (i.e. neurology, rehabilitation, medical, surgical, and otorhinolaryngology departments) during data collection were screened.

### Participants

Inclusion criteria were patients more than 18 years old, diagnosed with a stroke and ability to communicate in Bahasa Malaysia. Patients with traumatic intracranial haemorrhage, subdural haemorrhage, transient ischaemic attack, end-stage renal failure, severe psychiatric diseases, cancer, pregnancy or with amputation were excluded from the study. Patients with severe language or cognitive impairment were only included if a proxy of the patient was available. This proxy patient should be the primary caregiver of the actual patient at home and be familiar with the patient’s medical condition and lifestyle practices. All eligible participants were assessed and interviewed by a trained dietitian using a validated survey during the waiting period in outpatient clinics.

### Sample Size

The sample size of the present study was based on a single proportion formula, 95% confidence interval, 10% attrition rate, and a precision of ± 0.05 (24). Gomes et al. (12) reported that the prevalence of medium to high risk of malnutrition using a validated nutritional screening tool among stroke patients admitted to the stroke units in South London was 36%. Using Gomes et al.’s study as the reference, 395 patients were estimated to be required in the present study.

### Dependent Variable and Measurement

The dependent variable of the present study was the risk of malnutrition, which was assessed using the total scores of five indicators in the Malnutrition Risk Screening Tool-Hospital (MRST-H) scale (25–26). The first three indicators involved questions regarding financial dependency, feeding dependency and unintentional weight loss. The other two indicators included measurements of mid-upper arm circumference and calf circumference of the non-paralytic limb. A respondent was classified as having high malnutrition risk (≥ 2 scores) or low malnutrition risk (< 2 scores) based on the scores in MRST-H scale (25–26). The MRST-H scale has been validated among patients in the medical department (both inpatient and outpatient) hospital setting (25–26) in previous local studies, with excellent overall diagnostic accuracy and reliability. The MRST-H scale had 67% sensitivity and 90% specificity (25). The MRST-H scale has an excellent overall diagnostic accuracy in discriminating the malnourished group with area under the curve (AUC) of 0.84 when validated against the reference standard, Subjective Global Assessment (25). In addition, the MRST-H scale has good reliability with the Kappa index of agreement between dietitian with nurse A (81.3%, Kappa = 0.84) and nurse B (87.5%, Kappa = 0.89) (26).

### Independent Variables and Measurement

The independent variables in the present study included sociodemographic characteristics, clinical profiles, nutrition-related issues, BMI, dietary intake and hand grip strength. Demographic information and data on clinical characteristics of patients were obtained by reviewing the medical records and via interview-based surveys. This information included age, sex, ethnicity, marital status, education level, working status, monthly household income, number of comorbid conditions (hypertension, hyperlipidemia, diabetes mellitus, atrial fibrillation, and ischaemic heart disease), types of stroke (ischaemic or haemorrhagic stroke), duration of a stroke (months), number of episodes of stroke and smoking status. Additional questions regarding nutrition issues (Yes or No) were asked; these included the presence of chewing difficulties, loss of appetite, aphasia, paresis of the dominant arm, constipation, wheelchair-bound and tube feeding. The weight and height of patients were measured using a Tanita Model BC-541 digital weighing scale and Seca 213 portable stadiometer, respectively. Two readings were obtained, and the mean value was calculated. The BMI was calculated by dividing body weight (kg) with height (m^2^).

Participants’ habitual diet intake for the past 7 days at home was assessed based on the Dietary History Questionnaire (27). The participants with or without the assistance of caregivers were asked to recall all food and beverages usually consumed in a 1-week duration. Nutritionist Pro™ Nutrition Analysis tool was used for dietary analysis. For tube-fed participants, the brands of feeding powder, the number of scoops, dilutions and frequency of feeding were recorded. The mean of nutrient intakes, such as energy, carbohydrates, proteins, fats, iron, calcium, sodium, potassium, vitamin C, vitamin A, vitamin B1, vitamin B2, and vitamin B3 was calculated. The hand grip strength was measured using the Takei Digital Grip Strength Dynamometer, model T.K.K.5401, following the standard guidelines provided in the user manual. The hand grip measurement was repeated twice on the non-paralysed hand, either in the standing or seated position.

### Data Analysis

Data were analysed using the IBM SPSS version 25.0 for Windows. The normality of data was assessed using the Kolmogorov-Smirnov test. The normally distributed continuous variables were presented as mean and standard deviation, whereas non-normally distributed data were presented as median and interquartile ranges. The number and proportions were reported for categorical variables. The differences between high-risk and low-risk malnutrition groups were analysed using Student’s *t*-test or Mann-Whitney U-test for continuous variables. Chi-square test or Fisher’s exact test was used to compare the categorical variables. Crude odds ratios (ORs) and adjusted odds ratios (AORs) with 95% CI were reported in univariable and multivariable logistic regressions, respectively. The dependent variable in the study was malnutrition risk, whereas independent variables were sociodemographic characteristics, clinical profiles, anthropometric status and dietary nutrients intake. Multivariable logistic regression analysis was performed to examine predictors of malnutrition risk by including variables with *P*-value < 0.25 in the univariable analysis. Correlations and multicollinearity between the variables were checked and *P*-values < 0.05 were considered significant.

## Results

A total of 398 respondents were successfully recruited in the study with a mean age of 59.4 years. The majority of the participants were male, of Malay ethnicity, married, had obtained secondary education and above, were unemployed, and had a monthly household income less than RM2,000. Most of the participants presented with first ever stroke (81%), ischaemic type (71%) and with a mean duration of stroke of 28 months. More than half of the participants had two to three comorbidities (72%) and 51% were diagnosed with diabetes mellitus. One-fifth of the participants were wheelchair-bound and approximately 7% of them were active smokers during the survey. A small proportion (6%) of the participants were on complete tube feeding or mix feeding (oral + tube) during the data collection period. Tables 1 and 2 compare the demographic and clinical characteristics between high-risk malnutrition and low-risk malnutrition groups.

The present study revealed that approximately 40% of respondents were at a risk of malnutrition. After further examination of the BMI status for the high-risk group (not shown), it was found that 20% of them were underweight (BMI < 18.5 kg/m^2^), 36% had normal weight (BMI: 18.5–22.9 kg/m^2^) and 44% of them were overweight and obese (BMI ≥ 27.5 kg/m^2^). The mean BMI of all participants was 25.0 kg/m^2^. In addition, the high-risk malnutrition group consumed significantly lower daily energy (1,421 kcal versus 1,729 kcal/day), carbohydrates (190 g versus 238 g), proteins (57 g versus 65 g), fat (47 g versus 57 g), iron (13 g versus 15 g), sodium (1652 mg versus 2102 mg) and potassium (1,268 mg versus 1,358 mg) intake compared with the low-risk group (Table 3).

The univariable logistic analysis data presented in Table 4 shows that participants who attended the primary school level and lower, were unemployed, wheelchair-bound, and with monthly household income of less than RM2,000 were at higher odds of developing the risk of malnutrition. In addition, those who complained about nutritional issues, such as loss of appetite, aphasia, chewing difficulties, and were on tube feeding, were more likely to fall under the high-risk malnutrition group. Higher BMI, higher hand grip strength and intake of dietary nutrients, such as energy, carbohydrates, proteins, fats, iron, sodium and potassium, were significantly associated with lower odds of malnutrition risk.

The multivariable logistic analysis (Table 5) showed unemployment, tube feeding, loss of appetite, wheelchair-bound and BMI to be significant predictors of risk of malnutrition among stroke patients. The strongest predictor was tube feeding (AOR: 13.16, 95% CI: 3.22–53.77). In addition, patients who complained of loss of appetite (AOR: 8.15, 95% CI: 4.71–14.12) were eight times more likely to develop the risk of malnutrition. In contrast, wheelchair-bound patients (AOR: 2.23, 95% CI: 1.22–4.09) were twice more likely prone to the risk of malnutrition as compared to those who were able to ambulate. Moreover, an increase in BMI (AOR: 0.87, 95% CI: 0.82–0.93) was a protective factor for risk of malnutrition among stroke patients.

## Discussion

The present study showed that 40% of post-stroke patients were at a high risk of malnutrition, a finding similar to that reported by previous studies in Spain (46%) and France (48%) (19, 22). Yet, it was much lower than the 84% to 91% prevalence in two Korean studies (17–18). The discrepancy in results could be attributed to the methodological differences between the studies (e.g. elderly versus non-elderly, duration of stroke and nutritional screening instruments). Paquereau et al. (19) reported that 48% of stroke patients (with duration of stroke onset 1–5 years) were categorised as malnourished during admission to the neurologic rehabilitation unit in France. However, the study defined malnutrition based on the Mini Nutritional Assessment Short Form, BMI and presence of weight loss. In addition, it revealed that 40% of patients had significant weight loss, 38% of them had increased weight, whereas 21% of them maintained weight after 1 year (19). Furthermore, the prevalence of risk of malnutrition (40%) was almost four times higher than that reported (9%) based on BMI of less than 18.5 kg/m^2^. This was probably due to the fact that a large number of patients reported continuous unintentional weight loss during the survey (assessed by one of the indicators in MRST-H screening tools). Continuous unintentional weight loss or lean body mass is undesirable, especially for those in underweight or normal weight categories. Jönsson et al. (28) demonstrated that patients who had lost weight at 12 months after the stroke experienced more difficulties while eating and had lower pre-albumin levels. Undoubtedly, the prevalence of risk of malnutrition among post-stroke patients was high even in an outpatient setting. The lack of nutrition-related information in medical documents is another factor for high-risk malnutrition groups to remain undiagnosed and untreated (29–31). This addresses the need for continuous nutritional screening of stroke patients using a rapid and simple screening tool. Patients recognised as a high-risk group should be referred to experts (i.e. dietitians) for more detailed nutritional assessment and intervention.

Multivariate logistic regression revealed that the strongest predictor of malnutrition risk among stroke patients in the current study was tube feeding, a finding similar to that reported by previous studies conducted in acute setting (8, 15). The risk of tube-fed patients was 13 times higher than those fed orally. A case-control study among post-stroke elderly patients attending outpatient clinics in Malaysia demonstrated that the majority of patients (76%) on long-term tube feeding were moderately to severely malnourished (as assessed by Subjective Global Assessment) (32). The poorer nutritional status among tube-fed patients could be explained by the greater severity of the stroke and inadequate nutrient intake because of nasogastric tube complications (e.g. frequent tube dislodgement and delay in reinsertion) (32). In addition, tube feeding is indirectly indicative of the presence of dysphagia, very poor oral intake, and higher financial and social dependency on others.

In addition, patients who complained about loss of appetite were eight times more likely to develop the risk of malnutrition as compared to those who did not. This finding is similar to that of previous literature in both acute (8) and post-acute phase settings (19). Paquereau et al. (19) explained the risk of malnutrition among stroke survivors could be explained by the changes in food intake and preferences, such as reduced preferences for fatty and sweet food products. This might be related to brain lesions involved in sensing the taste of food. ‘Gourmand syndrome’ and loss of appetite have been reported among patients with right anterior lesions (33) and dorsomedial thalamic infarct (34), respectively. In addition, although inconsistent results have been reported, the role of depressed mood or anxiety after a stroke on dietary intake and appetite should not be ignored (19, 21). Therefore, it is important to closely monitor nutrition-related problems and investigate the reasons for appetite loss to ensure implementation of proper strategies to prevent malnutrition.

Further, the present study showed socioeconomic status determinants such as income, education, and employment status to be associated with increased odds of malnutrition risk, similar to the findings of a study in Spain (22). However, only employment status remained significant in the multivariate logistic analysis. Similarly, Paquereau et al. (19) observed no significant associations between income and education level with a long-term change over time in weight and risk of malnutrition. Although functional status or stroke severity was not assessed in the present study, we believe that patients who are able to work may suffer from a less severe stroke. Therefore, they may have better functional status and independence as compared to those who are unable to work. Functional status has been significantly associated with the risk of malnutrition in previous studies (17, 22).

In agreement with the findings of a previous study (8), the present study reported a higher risk of malnutrition among patients presenting with impaired mobility. Approximately 35% of patients in the high-risk malnutrition group were wheelchair-bound and, thus, highly dependent on other people for daily activities and food preparation. Moreover, it was observed that a large number of wheelchair-bound patients suffered from muscle wasting as indicated by the calf circumference, which was part of the indices in the MRST-H screening tool. Stroke-induced muscle abnormalities (i.e. denervation, disuse, remodelling and spasticity), together with prolonged immobility, could lead to decreased muscle protein synthesis and leg lean mass, resulting in reduced muscle strength (35, 36).

Unsurprisingly, BMI was significantly associated with the risk of malnutrition, which corroborated a previous result (15). Stroke patients with a higher value of BMI were 13% less likely to develop the risk of malnutrition. However, during the survey, it was found that a large proportion of patients had balance and gait issues, making the accurate measurement of weight and height difficult. Therefore, an alternative measurement (knee height and mid-upper arm circumference) (37) and predictive equation were required, which could indirectly demand longer time duration and higher skills displayed by clinical nursing or the medical staff (38). Considering the significant association between BMI and challenges in anthropometric measurements of stroke patients, a validated nutrition screening tool can be suggested as a rapid alternative method to assess the nutritional status among stroke patients before they are referred to appropriate experts for further assessment.

In terms of dietary nutrient intake, the present study revealed that post-stroke patients generally consumed an average of 1,606 kcal/day or 25.6 kcal/kg and 62 g protein/day, which were approximately 80% and 110% of the recommended nutrient intake (RNI) for Malaysia values, respectively. These findings are in line with those of a previous study in the United Kingdom (20) (i.e. 1,625 kcal/day and 63 g protein/day; otherwise 80% of the recommended estimated average requirement values for energy) but lower than those reported in Taiwan (39) (i.e. 1,714 kcal/day). In addition, the present study reported a lower percentage of RNI for energy intake among those in the high-risk malnutrition group (72% energy; 117% protein) compared to those in the low-risk malnutrition group (87% energy; 101% protein). This was in agreement with previous studies indicating that insufficient energy intake was more common than suboptimal protein intake among stroke patients (16, 17, 20). The significant lower intakes of energy, macronutrients (i.e. carbohydrates, proteins and fats), and micronutrients intake (i.e. iron, sodium and potassium) among patients in the high-risk malnutrition group warrant future studies to examine the causality factors. A possible explanation of lower nutrient intake among patients in the high-risk malnutrition group could be a combination of physical impairments and psychosocial issues, such as dysphagia, wheelchair-bound, upper arm paresis, fatigue, depression, loss of appetite, poor eating environment and presence of a recent acute illness.

Since the present study was an observational work, a causal relationship was, thus, difficult to establish. However, this was probably the first multicenter observational study with a relatively large sample size that examined the prevalence and predictors of malnutrition risk, as well as dietary nutrient intake among post-stroke patients in Malaysia. Therefore, it is believed that the results generated from this study will contribute important information regarding nutrition issues among stroke patients. In addition, only patients attending outpatient clinics in hospital settings were included in this work. These patients tend to have more severe impairments and disability as compared to those having a follow-up appointment in primary health clinics or those who were discharged. This may have contributed to the relatively high prevalence risk of malnutrition. Several studies have described the energy and protein intake of stroke patients during acute admission or in the rehabilitation ward (14, 40–45). However, to the best of our knowledge, the present work is probably one of the few that compared nutrient intakes (i.e. macro- and micronutrients) of post-stroke patients in an outpatient setting using the malnutrition risk classification.

## Conclusion

The risk of malnutrition was highly prevalent among post-stroke patients and was significantly associated with tube feeding, loss of appetite, unemployment, wheelchair-bound state and BMI. Based on these findings, early screening and proper treatment of malnutrition are highly recommended during the acute phase and follow-up appointments in primary clinics and hospital settings.

## Figures and Tables

**Table 1:** Comparison of sociodemographic characteristics between low-risk and high-risk malnutrition groups (*n* = 398)

Variables	Low-risk malnutrition (*n* = 239) *n* (%)	High-risk malnutrition (*n* = 159) *n* (%)	*X*^2−^statistic (df)	*P*-value^a^
Sex			0.16 (1)	0.693
Female	107 (44.8)	68 (42.8)		
Male	132 (55.2)	91 (57.2)		
Ethnicity			0.31 (2)	0.857
Malay	209 (87.4)	136 (85.5)		
Chinese	25 (10.5)	19 (11.9)		
Indian	5 (2.1)	4 (2.6)		
Age (years)			6.42 (3)	0.087
20–39	11 (4.6)	7 (4.4)		
40–59	110 (46.0)	59 (37.1)		
60–79	116 (48.5)	87 (54.7)		
≥ 80	2 (0.9)	6 (3.8)		
Marital status				
Married	180 (75.3)	112 (70.4)	1.16 (1)	0.281
Single/widowed/divorced	59 (24.7)	47 (29.6)		
Education level				
Primary or less	68 (28.5)	69 (43.4)	9.83 (2)	0.007
Secondary	141 (59.0)	77 (48.4)		
Tertiary	30 (12.5)	13 (8.2)		
Employment status				
Employed	42 (17.6)	7 (4.4)	16.05 (1)	<0.001
Unemployed	197 (82.4)	152 (95.6)		
Monthly income (RM)				
< 1,000	78 (32.6)	84 (52.8)	23.42 (6)	0.001
1,000–1,999	66 (27.6)	43 (27.0)		
2,000–2,999	29 (12.1)	12 (7.5)		
3,000–3,999	18 (7.5)	8 (5.0)		
4,000–4,999	14 (5.9)	4 (2.5)		
5,000–5,999	6 (2.5)	3 (1.9)		
≥ 6,000	28 (11.8)	5 (3.3)		

Notes: RM1 = USD0.24

a Chi-square test for independence with significance level *P* < 0.05

**Table 2:** Comparison of clinical profiles and nutrition-related issues between low-risk and high-risk malnutrition groups (*n* = 398)

	Low-risk malnutrition (*n* = 239) *n* (%)	High-risk malnutrition (*n* = 159) *n* (%)	*X^2^*^−^statistic (df)	*P*-value^a^
Duration of stroke (months) (mean) (SD)	32.1 (43.5)	22.8 (48.3)	2.00 (396)	0.046^b^
Stroke episodes				
First	198 (82.8)	125 (78.6)	1.22 (2)	0.543
Second	36 (15.1)	29 (18.2)		
Third	5 (2.1)	5 (3.2)		
Types of stroke				
Ischaemic	165 (69.0)	119 (74.8)	2.47 (2)	0.290
Haemorrhagic	51 (21.3)	31 (19.5)		
Unspecified	23 (9.7)	9 (5.7)		
Number of co-morbid				
0–1	23 (9.6)	18 (11.3)	0.75 (2)	0.689
2–3	192 (80.4)	122 (76.7)		
≥ 4	24 (10.0)	19 (12.0)		
Diabetes mellitus				
Yes	115 (48.1)	89 (56.0)	2.36 (1)	0.125
No	124 (51.9)	70 (44.0)		
Chronic kidney disease				
Yes	26 (10.9)	14 (8.8)	0.45 (1)	0.500
No	213 (89.1)	145 (91.2)		
Smoking status				
Never smoke	150 (62.8)	94 (59.1)	1.14 (2)	0.567
Ex-smoker	71 (29.7)	55 (34.6)		
Smoking	18 (7.5)	10 (6.3)		
Wheelchair-bound				
Yes	37 (15.5)	71 (44.7)	41.10 (1)	< 0.001
No	202 (84.5)	88 (55.3)		
Route of feeding				
Oral	236 (98.7)	137 (86.2)	25.67 (1)	< 0.001
Tube feeding/mix	3 (1.3)	22 (13.8)		
Paresis at dominant arm				
Yes	129 (54.0)	95 (59.7)	1.29 (1)	0.255
No	110 (46.0)	64 (40.3)		
Loss of appetite				
Yes	41 (17.2)	89 (56.0)	65.42 (1)	< 0.001
No	198 (82.8)	70 (44.0)		
Aphasia/slurred speech				
Yes	65 (27.2)	62 (39.0)	6.12 (1)	0.013
No	174 (72.8)	97 (61.0)		
Chewing difficulty				
Yes	20 (8.4)	45 (28.3)	27.77 (1)	< 0.001
No	219 (91.6)	114 (71.7)		
Constipation				
Yes	97 (40.6)	79 (49.7)	3.61 (1)	0.056
No	142 (59.4)	80 (50.3)		

Notes: Comorbidities included hypertension, hyperlipidemia, diabetes mellitus, atrial fibrillation, and ischaemic heart disease.

a Chi-square test for independence with significance level *P* < 0.05;

b Independent *t*-test

**Table 3:** Comparison of dietary nutrient intake and anthropometric indices between low-risk and high-risk malnutrition groups (*n* = 398)

Variables	Low-risk malnutrition (*n* = 239) mean (SD)	High-risk malnutrition (*n* = 159) mean (SD)	*t*-statistic (df)	*P*-value^a^
Body mass index (kg/m^2^)	26.6 (4.7)	22.8 (4.7)	8.00 (396)	< 0.001
Energy (kcal)	1729.1 (369.9)	1420.8(343.8)	8.37 (396)	< 0.001
Energy/kg (kcal/kg)	25.9 (3.9)	25.2 (5.5)	1.42 (262)	0.157
Protein (g)	65.0 (13.5)	56.6 (14.7)	5.87 (396)	< 0.001
Carbohydrate (g)	238.4 (56.0)	189.9 (52.1)	8.70 (396)	< 0.001
Fat (g)	56.9 (16.8)	47.0 (16.0)	5.84 (396)	< 0.001
Iron (mg)	15.2 (5.4)	13.1 (5.2)	3.86 (396)	< 0.001
Vitamin A (RE)	799.4 (370.0)	754.6 (424.9)	1.12 (396)	0.266
Calcium (mg)	658.1 (321.6)	665.2 (360.9)	−0.21 (396)	0.836
Thiamine (mg)	0.8 (0.4)	0.9 (0.6)	−1.00 (254)	0.318
Riboflavin (mg)	1.3 (0.5)	1.3 (0.6)	1.17 (292)	0.243
Niacin (mg)	10.9 (5.0)	11.5 (6.8)	−0.95 (270)	0.343
Vitamin C (mg)	102.1 (56.9)	108.9 (63.6)	−1.13 (396)	0.261
Sodium (mg)	2101.6 (686.8)	1652.0 (614.4)	6.67 (396)	< 0.001
Potassium (mg)	1357.5 (393.7)	1268.0 (444.8)	2.11 (396)	0.036
Hand grip strength (kg)	23.62 (9.40)	18.42 (9.07)	5.49 (396)	< 0.001

Notes: kg/m^2^ = kilogram/meter^2^; kcal = kilocalories; g = gram; mg = milligram; RE = retinol equivalent; kg = kilogram

a Independent *t-*test with significance level *P* < 0.05

**Table 4:** Associated factors of malnutrition risk with univariable logistic regression (*n* = 398)

Variables	b	Crude odd ratio (95% CI)	*P*-value^a^
Education level			
Tertiary^b^		1.00	
Secondary	0.23	1.26 (0.62, 2.56)	0.522
Primary or less	0.85	2.34 (1.13, 4.87)	0.023
Employment			
Employed^b^		1.00	
Unemployed	1.56	4.63 (2.02, 10.59)	< 0.001
Wheelchair-bound			
No^b^		1.00	
Yes	1.48	4.41 (2.75, 7.05)	< 0.001
Route of feeding			
Oral^b^		1.00	
Tube feeding/mix	2.54	12.63 (3.71, 43.0)	< 0.001
Monthly income (RM)			
≥ 6,000^b^		1.00	
< 1,000	1.80	6.03 (2.22, 16.40)	< 0.001
1,000–1,999	1.29	3.65 (1.31, 10.18)	0013
2,000–2,999	0.84	2.32 (0.72, 7.43)	0.158
3,000–3,999	0.91	2.49 (0.70, 8.81)	0.158
4,000–4,999	0.47	1.60 (0.37, 6.91)	0.529
5,000–5,999	1.03	2.80 (0.52, 15.04)	0.230
Body mass index (kg/m^2^)	−0.19	0.83 (0.79, 0.87)	< 0.001
Energy (kcal)	−0.003	0.997 (0.997, 0.998)	< 0.001
Protein (g)	−0.043	0.958 (0.943, 0.973)	< 0.001
Carbohydrate (g)	−0.017	0.983 (0.978, 0.987)	< 0.001
Fat (g)	−0.039	0.962 (0.948, 0.975)	< 0.001
Iron (mg)	−0.078	0.925 (0.888, 0.964)	< 0.001
Sodium (mg)	−0.001	0.999 (0.999, 0.999)	< 0.001
Potassium (mg)	−0.001	0.999 (0.999, 1.00)	0.037
Hand grip strength (kg)	−0.061	0.94 (0.92, 0.96)	< 0.001
Loss of appetite			
No^b^		1.00	
Yes	1.82	6.14 (3.88, 9.72)	< 0.001
Aphasia/slurred speech			
No^b^		1.00	
Yes	0.54	1.71 (1.12, 2.62)	0.014
Chewing difficulties			
No^b^		1.00	
Yes	1.46	4.32 (2.44, 7.67)	< 0.001

Notes: RM1 = USD0.24

a Likelihood ratio test with significance level set at less than 0.05;

b Reference category

**Table 5:** Associated factors of malnutrition risk with multivariable logistic regression (*n* = 398)

Variables	Adjusted b	AOR (95% CI)^a^	*P*-value^b^
Employment			
Employed^c^		1.00	
Unemployed	1.47	4.26 (1.64, 11.12)	0.003
Wheelchair-bound			
No^c^		1.00	
Yes	0.80	2.23 (1.22, 4.09)	0.009
Route of feeding			
Oral^c^		1.00	
Tube feeding/mix	2.58	13.16 (3.22, 53.77)	< 0.001
Body mass index (kg/m^2^)	−0.14	0.87 (0.82, 0.93)	< 0.001
Loss of appetite			
No^c^		1.00	
Yes	2.10	8.15 (4.71, 14.12)	< 0.001

Notes:

a Adjusted for household income, energy, protein, carbohydrate, fat, iron, sodium, potassium, hand grip strength, presence of aphasia, and chewing difficulties;

b Likelihood ratio test with significance level set at less than 0.05;

c Reference category Multicollinearity and interaction terms were checked and not found; Hosmer-Lemeshow test, (*P* = 0.145), classification table (overall correctly classified percentage = 79.9%) and area under the receiver operating characteristic (ROC) curve (84.7%) were applied to check the model fitness
